# Intelligence, &c.

**Published:** 1827-04

**Authors:** 


### PRIZE HOSPITAL REPORTS.

In answer to several enquiries, nominal and anonymous, we beg to say, that the Candidates for the Prize are *not* confined to *Pupils* of Hospitals exclusively. The Reports must be authenticated by the name of the Reporter. This is the only absolute condition. For reasons which will appear in our next, two Prizes, *in succession*, cannot be adjudged to the same Hospital. The Reports must reach the Editor by the 1st of the Month preceding publication; viz. by the 1st of June—1st of September—1st of December, and 1st of March.

Two Prizes will be awarded for the ensuing Quarter—one of which, it is to be hoped, will be for a provincial Hospital Report. Each Report will be allotted a space varying from 32 to 48 *Columns*, such as the present Report is printed in.

### INTELLIGENCE, CORRESPONDENCE, &c.

*Chlorides of Soda and of Lime.*

Since this disinfecting and medicinal agent is likely to come into considerable demand, it will be gratifying to learn that it is be procured at a very cheap rate—no inconsiderable advantage! Mr. Fincham, an ingenious and scientific wholesale manufacturing chemist of Nottingham, has now furnished his London agents (especially Mr. Philp, of Regent Street) with abundance of these articles, which may now be procured at a very reasonable price indeed. We have no doubt that medical practitioners will forthwith supply themselves with these chlorides, and make trials of them for the various purposes which are amply detailed in the present number of this Journal.

*Medico-Chirurgical Society.*

On the 1st March, the following officers were elected for the ensuing year, viz. Mr. Travers, President—Dr. Merriman, Mr. Stanly, Dr. Bright, and Mr. Rose, Vice-Presidents—Dr. Roget and Mr. Earle, Treasurers—Dr. Seymour, and Mr. Eusebius Lloyd, Secretaries—Dr. J. Johnson and Mr. Arnott, Librarians. The other members of the Council are, Dr. Baker, Mr. Broughton, Dr. H. Davies, Mr. Money, Mr. Powell, Mr. Alcock, Dr. Tweedie, Mr. R. Welbank, Dr. Roberts, Mr. Ashwell.

The new President and Secretaries took their seats on the 13th March, when Mr. Travers delivered a very appropriate speech from the chair. He animadverted in delicate, but very forcible language, on the supineness of the more influential members of the society, during the last two years, by which supineness and negligence of attendance at the meetings, the character of the Society was, in a degree, compromised and lowered in public estimation. The president candidly acknowledged that he himself was among the delinquents, (if such a term is admissible) and did not impute any blame to others, in which he did not fully participate himself.

Indeed we cannot allow this opportunity to pass, without reiterating and enforcing the observation of the president, that, the grand (we would say the *sole*) cause of the decline of the Society is *apathy* among its members—and that it requires nothing more than the *presence* of the members at its meetings, to restore, at once, the society to all its pristine splendour and respectability. We conceive it to be a species of ingratitude in the higher orders of the profession, whose names are enrolled on its lists, to desert the society in the days of their own prosperity. They owe something to public opinion, to the junior members, and to the promotion of that harmony and kindly feeling among medical men, which associations of this kind are so well calculated to effect. The appeal which is now made to the members of the Medico-Chirurgical Society, will, we have no doubt, rouse them from their apathy, and render the meetings of this excellent institution, interesting to themselves, and its transactions advantageous to the profession at large. The first meeting, under the new president, augurs well. An important paper was read from Mr. Welbank, and much valuable information was elicited from Mr. Lawrence, Mr. Earle, Dr. A. T. Thomson, &c.

*(Circular.)* "In consequence of a correspondence which has taken place between the President of the College of Physicians, and the President of the College of Surgeons, the Board of Curators have adopted the following resolution."—"That the Licentiates of the College of Physicians shall hereafter be admitted to the Museum of the College of Surgeons, upon all days of public exhibition, without further ceremony than that of inscribing their names in the visitors' book."

*Obituary—Mr. Bampfield.*

We have deeply to regret the recent and premature death of this able and amiable surgeon, well known to his professional brethren by several works of sterling merit—especially his Treatise on Scorbutic Dysentery, and that on Diseases of the Spine. Mr. Bampfield had served many years as surgeon in the Royal Navy, and, at the conclusion of the late war, settled in Bedford-street, Covent-garden, as a general practitioner. There he may be said to have originated a medical practice, and to have brought it to a very considerable extent by his talents, his integrity, and his humane attention to the sick. He was universally beloved and respected by all who knew him, both in and out of the profession.

For many years past he laboured under some obscure affection of the chest, attended with cough and some dyspnœa, especially when ascending stairs, or walking fast. He did not, however, attach importance to his own complaint, and would never relax, on that account, from his professional labours. It was only about three months before his death, when labouring under some slight bronchial inflammation, which aggravated his original malady, that he sent for the Editor of this Journal, who, on examining the chest, immediately discovered the fatal nature of the disease. The heart was enlarged to a very considerable extent, and, that peculiar and dangerous whizzing, or purring noise was heard, during each ventricular contraction, which has been mentioned by Laennec and others, and of which we gave a remarkable instance at page 213 of our last Number. As we have always found this phenomenon indicative of fatal organic disease, the melancholy event was now anticipated by the reporter, though concealed from the patient. At a later period, Dr. Elliotson joined the Editor of this Journal, in an auscultic examination of the chest, and Dr. E. came to the same conclusion as Dr. Johnson did. Dropsical effusions took place, and were relieved by diuretics, &c. but the fatal malady made progress, and this excellent member of our profession died suddenly at Reading, whither he had gone for a few weeks, in hopes that change of air might mitigate his sufferings. Mr B. has been succeeded in his practice by Mr. Brookes, late of Reading, brother to Mr. Joshua Brookes the anatomist.

---

*Ouvrages Nouveaux qui se trouvent chez J. B. Baillière, 3, Bedford Street, Bedford Square, London.*

*Dutrochet.* L'Agent immediat du Mouvement Vital Dévoilé dans sa Nature et dans son Mode d'Action chez les Végétaux et chez les Animaux. Paris, 1827, en 8vo. Prix 4s.

*Calmeil.* De la Paralysie considerée chez les Aliénés; Recherches faites dans le Service de feu *M. Royer Collard* et de *M. Esquirol.* Paris, 1826, en 8vo. Prix 6s. 6d.

*Hurtrel d'Arboval.* Dictionnaire de Medecine et de Chirurgie Vétérinaires, tom. 1, en 8vo. Paris, 1826. Prix 16s. (Le Tom. 3 paraitra dans deux mois.)

Memoires de la Société Medical d'Emulation de Paris. Tom. 9, 8vo. Paris, 1826. Prix 8s. (Cet ouvrage est aussi publié sous le titre de tom. 1, pour les personnes qui n'ont pas le commencement de la colection.)

*Ratier et O. Henry.* Pharmacopeé Française, ou Code des Medicamens, nouvelle traduction du Codex Medicamentarius sive Pharmacopœa Gallica. Paris, 1827, en 8vo. prix 8s.

*Rayer.* Traité Theorique et Pratique des Maladies de la Peau, fondé sur de nouvelles Recherches d'Anatomie et de Physiologie Pathologique. Tom. 1, en 8vo. Et atlas de 10 planches gravés et coloriées, offrant plus de 60 variétés de maladies de la peau. Paris, 1827, prix 18s. (Le tom 2 et dernier paraitra en Avril.)

*Portal.* Observations sur la Nature et le Traitement de l'Epilepsie. En 8vo. prix 8s. Paris, 1827.

*Londe.* Nouveaux Elemens d'Hygiene redigés suivant les principes de la nouvelle Doctrine Medicale. Paris, 1827. 1 tom. en 8vo prix 12s.

*Tiedmann.* Tabula Arteriarum Corporis Humani. *Carlsruhe,* 1822 et 24. 1 vol. en 4to, de Text (Latinæ et Germanæ), et 76 Planches coloriées grand in fol atlantique relié. Prix 12l. 10s.

*J. Thomson.* Traité Medico-Chirurgical de l'Inflammation. Traduit de l'Anglais avec des notes par F. G Boisseau et A. T. L. Jourdan, un fort vol. en 8vo. Prix 9s. Paris.

*Roche et Sanson.* Nouveaux Elemens de Pathologie Medico-chirurgical, ou precis Theorique et Pratique de Medecine de Chirurgie, tom. 1 et 2, 2d vol. en 8vo.

Prix 17s. (Le tom. 3 et dernier, paraitra dans un mois.)

*Voisin* des Causes Morales et Physiques des Maladies Mentales, et quelques autres Affections Nerveuses, tellesque l'Hysterie, la Nymphomanie, et le Satyriasis. Paris, 1826. 1 vol. 8vo. prix 7s.

*Gall.* Sur les Fonctions du Cerveau et sur celles de chacune des ces Parties, &c. Paris, 1827. 6 vols. en 8vo. prix 2l. 2s.

---

The Editor has received a long letter from Dr. Barnes, of Carlisle, complaining of the review of his paper on "Vicarious Menstruation," published in the 4th No. of the Edinburgh Journal of Medical Science, and analyzed in the 11th No. of this Series, page 248. Dr. B. calls on the Editor, in warm language, to review the whole transaction, and compare the original with the critique, or, rather, analysis, in order to do justice between the reviewer and reviewed. Although the Editor has but little time for such revisals, he did compare the papers, and while he acknowledges that the reviewer of Dr. Barnes's paper has misunderstood him on some points, of no great importance, yet he must also take the liberty of saying, that *he also* had very great difficulty in connecting the statements, and making out the precise meaning of Dr. Barnes. The Editor cannot help observing, that Dr. Barnes appears to be a great deal too sensible to the *remarks* of the reviewer (for they really do not deserve the name of *criticisms*); and that there does not appear to be any thing in these remarks which throws the slightest doubt upon the accuracy of Dr. Barnes's statements, the correctness of his judgment, or the respectability of his professional attainments. The reviewer of Dr. Barnes's paper resides nearly 500 miles from Carlisle, and therefore could have no possible object or wish to misrepresent him. He has misunderstood him on some points; but his comments are so mild, that they really require no other observation than what is here stated. With this explanation the Editor hopes Dr. Barnes will be satisfied.

---

Mr. Lizars, in answer to some queries contained in our last Number, begs to inform the subscribers to his Anatomical Work, that he shall lose no time in writing the remainder of the Physiological and Pathological Observations.

*Cooper's Surgical Dictionary.*

A correspondent has suggested to us, that this valuable work might be converted into a System, as well as a Dictionary, by means of a table of its contents, arranged somewhat in the same way that Dr. Ure has done in his Dictionary of Chemistry. For instance, inflammation and its consequences might be placed under the first head of this tabular view; tumours, &c. in the second, and so on. Were this plan adopted in the new editions, we cannot help thinking, that it would be of great advantage, both to the student and more advanced branches of the profession.

---

We are sorry that, owing to a misapprehension of the printer, a notice re-appeared in our last Number, purporting that all the back Nos. of the Analytical Series, imported from America, were in readiness for delivery. The Journals have been twice procured from America, but there are none now remaining. Mr. Millar, of Bridge Street, Blackfriars, has been commissioned to procure a fresh supply, and as soon as they arrive, notice will be given to our subscribers.

---

The cases of poisoning, by Belladonna, received from our friend in the North, will appear in our next.

---

The Medical Botany will be regularly noticed in future, as the numbers come out. In the mean time, we strongly recommend the work to the medical profession.

---

*Sound Chirurgical Knowledge.*

We deem it necessary to notice a most unique article in the Lancet of March 17th, on a critique or two which appeared in our last number. If our readers will have the goodness to turn to page 195, of that number, they will find a pretty full exposé of a case which appeared in the Lancet, as a varicose aneurism of the *radial* artery. We stated that we were quite sure that it was no varicose aneurism at all, and we *supposed*, from the manner in which the case was drawn up, that the *brachial*, and not the *radial* artery was wounded. After nearly three months preparation, a reply has been sent forth, and from the *talent* which it exhibits, we should have concluded it was the production of the reporter himself, did not the *characteristic* elegance of style, at once stamp its author.

"Let us examine (says the Lancet) what he has produced. First there is a note of interrogation affixed to the words '*radial* artery;' and he goes on to say, '*we* suppose the *brachïal* was meant;' 'no high bifurcation mentioned;' thereby intimating, that our reporter did not know whether the brachial or radial artery was wounded. The '*Scribe*' should recollect, that a *dissection* of the limb had been made previous to the writing of the report; that the *radial artery* was *found* to be *wounded*, and, *par consequence*, it must be given off 'higher up than usual,' which is no uncommon thing. Was it necessary to tell *our* readers, that if the *radial* artery were wounded *at the bend of the fore-arm*, it must have have had '*a high division?*' To have done so, would have been an outrage on their judgment. But he does not say that the *radial* was *not* the artery *wounded*. Oh! no. He knows that it *was*."—*Lancet*, 770.

It would have been an insult, it seems, for this writer to have told *his* readers "that if the *radial* artery were wounded *at the bend of the fore-arm*, it must have *had a high division*." Oh yes; but this same person thinks it no insult to tell his readers what is utterly untrue. There was NO HIGH DIVISION—the RADIAL ARTERY WAS NOT WOUNDED. The dissection was made by Mr. Alcock—the *preparation* is at Mr. Guthrie's theatre, in Warwick-street; shewn at his lectures, seen by his pupils. The wound *is* in the BRACHIAL artery. This is fact—this is proof. But what of that? Facts and proofs are to the Lancet—as snow to the fire.

Take now a specimen of sound chirurgical knowledge.

"If" says he "he did, (know any thing of aneurism) 'he would not have ranked 'circumscribed aneurism,' and 'aneurismal varix,' and 'varicose aneurism,' as *three distinct* consequences of wounding an artery in phlebotomy. *Aneurismal varix*, and *varicose aneurism*, being *synonimous* terms for a pulsating tumour formed by the flowing of arterial blood into a suprajacent vein, in consequence of a communication established between them, by the vein having been transfixed, and the artery punctured, as occasionally happens in venesection. Such aneurism may be either *circumscribed*, or *diffused*, (in consequence of the escape of some arterial blood into the surrounding cellular tissue,) but it is no less an aneurismal varix, or varicose aneurism, on that account."—*Lancet*, 771.

It is not? Why the very class-books of the schools confute this. Take, for instance, Harrison on the Arteries, one of the latest and the best.

"Thus from accident," says he, "in venesection, four forms of aneurism may arise; first, circumscribed aneurism, filling up the hollow at the bend of the elbow; secondly, diffused aneurism, in which the disease extends from the elbow along the line of the artery towards the axilla; in both these forms of the disease, the operation of tying the brachial artery may be necessary; thirdly, *aneurismal varix*; and fourthly, varicose aneurism: in neither of which will an operation be generally required, except under the circumstances above-mentioned," namely, "in some cases of varicose aneurism, in which the intermediate sac has increased in size, and compressing the vein has extended itself as a common aneurismal tumour so as to require similar treatment." We might refer to authors innumerable, to the same effect, if we chose, but we have neither space, nor is it worth the trouble, for the merest tyro knows the fact. So much then for the veracity and surgical acumen of the *matter*; as for the beauties and elegancies of the *manner*, we have not courage to grace our pages with specimens of *them*.

The base insinuation, that we have ever thrown out imputations against any class of our professional brethren, we repel with scorn. The *object* of such insinuation is so manifest, that it will be seen at a glance by the most superficial observer. *Our object* is not to sow discord among the members of the medical profession, and by setting them in arms against each other, to degrade them from the rank of a liberal and enlightened faculty. No! Our wish is to promote harmony—and our labours have ever been directed to this end, as well as to the diffusion of useful knowledge, among all ranks of the profession. For the truth of these assertions, we appeal to all those who have perused the pages of this Journal from its commencement.

